# Mineral Intake and Cardiovascular Disease, Cancer, and All-Cause Mortality: Findings from the Golestan Cohort Study

**DOI:** 10.3390/nu16030344

**Published:** 2024-01-24

**Authors:** Mohammad Hosein Yazdanpanah, Maryam Sharafkhah, Hossein Poustchi, Arash Etemadi, Mahdi Sheikh, Farin Kamangar, Akram Pourshams, Paolo Boffetta, Sanford M. Dawsey, Christian C. Abnet, Reza Malekzadeh, Maryam Hashemian

**Affiliations:** 1 Liver and Pancreatobiliary Diseases Research Center, Digestive Diseases Research Institute, Tehran University of Medical Sciences, Tehran 1461884513, Iran; mhyazdanpanah@yahoo.com (M.H.Y.); msharafkhah@gmail.com (M.S.); h.poustchi@gmail.com (H.P.); akrampourshams@gmail.com (A.P.); 2 Metabolic Epidemiology Branch, Division of Cancer Epidemiology and Genetics, National Cancer Institute, National Institutes of Health, Bethesda, MD 20892, USA; arash.etemadi@nih.gov (A.E.); dawseys@dcpcepn.nci.nih.gov (S.M.D.); abnetc@mail.nih.gov (C.C.A.); 3 Genomic Epidemiology Branch, International Agency for Research on Cancer, 69366 Lyon, France; sheikhm@iarc.who.int; 4 Department of Biology, School of Computer, Mathematical and Natural Sciences, Morgan State University, Baltimore, MD 21251, USA; farin.kamangar@morgan.edu; 5 Stony Brook Cancer Center, Stony Brook University, Stony Brook, NY 11794, USA; paolo.boffetta@stonybrookmedicine.edu; 6 Department of Medical and Surgical Sciences, University of Bologna, 40126 Bologna, Italy; 7 Digestive Oncology Research Center, Digestive Diseases Research Institute, Tehran University of Medical Sciences, Tehran 1411713135, Iran; 8 Epidemiology and Community Health Branch, Division of Intramural Research, National Heart, Lung and Blood Institute, National Institutes of Health, Bethesda, MD 20892, USA

**Keywords:** mortality, cardiovascular disease, cancer, dietary mineral intake, Iran

## Abstract

Associations between mineral intake and mortality in non-Western countries have not been studied adequately. This study evaluated these associations in the Golestan Cohort Study, featuring a Middle Eastern population. The mineral intake was estimated from the baseline food frequency questionnaire, adjusted by using the nutrient density method, and divided into quintiles. We used Cox proportional hazards models to estimate hazard ratios (HR) and 95% confidence intervals (CI) for the mortality. We analyzed 41,863 subjects with a mean age of 51.46 ± 8.73 years at the baseline. During 578,694 person-years of follow-up (median: 14.1 Years), 7217 deaths were recorded. Dietary calcium intake was inversely associated with the all-cause mortality (HR_Q5 vs. Q1_ = 0.91, 95%CI = 0.85–0.99). We observed significant associations between calcium (HR_Q5 vs. Q1_ = 0.82, 95% CI = 0.73–0.93), copper (HR_Q5 vs. Q1_ = 1.11, 95% CI = 0.99–1.26), and selenium intake (HR_Q5 vs. Q1_ = 1.14, 95% CI = 1.01–1.29) and CVD mortality. Dietary phosphorus (HR_Q5 vs. Q1_ = 0.81, 95%CI = 0.69–0.96) and copper intake (HR_Q5 vs. Q1_ = 0.84, 95%CI = 0.71–0.99) were inversely associated with cancer mortality. In this study within a Middle Eastern population, a higher dietary intake of calcium exhibited an inverse association with all-cause mortality. Furthermore, nuanced associations were observed in the cause-specific mortality, suggesting potential avenues for dietary interventions and emphasizing the importance of considering dietary factors in public health strategies.

## 1. Introduction

Noncommunicable diseases (NCD), such as cardiovascular disease (CVD) and cancer, are the world’s leading causes of death. CVDs account for the most NCD deaths, with 17.9 million people annually, followed by various cancers (9.3 million). Of all NCD deaths, 77% take place in low- and middle-income countries [1]. More than 2.8 million people died from NCDs in the Eastern Mediterranean region in 2019 [2].

Modifiable lifestyle risk factors, such as unhealthy nutrition, inactivity, obesity, being overweight, smoking, and excessive alcohol consumption are associated with major NCDs (cancers and cardiovascular diseases) [3,4,5]. Micronutrient deficits are a prevalent dietary issue in the general population, and they are more common in individuals who have comorbidities [6]. These flaws will significantly impact current and future health outcomes and healthcare expenditures.

In a study of 30,899 US adults, it has been suggested that a lower all-cause mortality is associated with an adequate intake of magnesium and also that a lower CVD mortality is associated with adequate intakes of zinc and copper from foods [7]. In a sizeable Pan-European cohort, without taking into account lifestyle, sociodemographic characteristics, or other established dietary risk factors, the micronutrient sufficiency of diets was inversely correlated with the overall and cause-specific death rates [8]. Still, there is a very limited number of studies that evaluated the associations between mineral intake and CVD and cancer mortality in Middle Eastern countries with a different diet. Understanding the Middle East’s unique context allows us to compare and contrast findings with other regions, providing a broader perspective on the relationship between dietary mineral intake and mortality. Also, prior research has revealed the high prevalence of mineral deficiencies within the Golestan Cohort Study (GCS), in Iran, the most extensive cohort study in the Middle East. This extensive dataset offers a valuable opportunity to investigate the correlation between mineral deficiencies and their impact on health.

We aimed to study the association of element intakes such as calcium, zinc, iron, magnesium, phosphorus, potassium, copper, manganese, and selenium with all-cause and cause-specific mortality in the GCS.

## 2. Materials and Methods

### 2.1. Study Population

The GCS was conducted in north-eastern Iran to primarily investigate the potential risk factors of upper gastrointestinal cancers; however, all major causes of death were also investigated. Details on the research methodology have already been published [9]. Around 50,000 individuals, mostly rural populations, between the ages of 40 and 75 were enlisted from 2004 to 2008.

At the baseline, written informed consent was provided and a food frequency questionnaire (FFQ) was completed. Also, trained interviewers utilized a general questionnaire to gather baseline information on demographic characteristics, residential history, marital status, ethnicity, education, medical history, and lifestyle habits such as opium usage, cigarette use, and alcohol use. Height and weight were also measured. We calculated the body mass index (BMI) by dividing the weight of a participant (in kg) by the square of their height (in m^2^).

In this longitudinal study, all subjects of the GCS with available data on their FFQ were entered into the study. Those with missing data on baseline characteristics and previous medical conditions (cancer, heart disease, a previous stroke, and diabetes) at the baseline and subjects with less than 2 years of follow-up data were excluded. Also, participants reporting extreme intakes of total energy (energy intake is more than two interquartile ranges above the 75th or below the 25th percentile of energy intake) and participants who had more than 30 items missing in the FFQ were excluded (total exclusion = 7310) (Figure S1).

Physical activity during work and leisure time were utilized to calculate the metabolic equivalent of each task [10]. Based on home furnishings, vehicles, and other wealth-related data, a multiple correspondence analysis was employed to determine a wealth score. The classification of wealth scores and physical activity was made in tertiles. Single or married were the two categories for marital status. People who had been divorced or widowed were rare; thus, they were grouped as single. We categorized regular opium users as individuals who had consumed opium at least once a week for a minimum duration of 6 months. The Digestive Diseases Research Institute’s ethical review committee (Ref: FWA00001331), the International Agency for Research on Cancer (Ref: CN/23/3), and the US National Cancer Institute approved the study protocol.

### 2.2. Dietary Intake

We utilized a 116-item semiquantitative FFQ at the baseline to evaluate dietary intake. Prior research on this population indicated that the FFQ was valid and reliable [11]. This population predominantly follows a traditional dietary pattern. The participants were asked to describe their frequency of consumption of each food item from a list of foods with standard serving sizes on a daily, weekly, or monthly basis, over the preceding year. The portion sizes were converted from units of measurement to grams. By multiplying each food’s daily intake by its nutrient content, the nutrient intakes were estimated [12]. The nutrient density model was used to adjust the mineral intakes for energy intake [13]. In this region, supplement use is uncommon; hence, only mineral intakes from food sources were included.

### 2.3. Outcome

Every year, we followed up with each participant by phone. Family, friends, or local healthcare providers reported deaths in the participants, and a team visited their homes to identify any reported deaths by completing a verbal autopsy questionnaire that had been validated for this population [14]. Available relevant medical records, such as medical charts, radiographs, pathology reports, hospital discharge records, etc., were collected from the appropriate hospitals or pathology centers, either in the province or in neighboring provinces. All the records were evaluated, and the International Classification of Diseases, Tenth Revision (ICD-10), was used to determine the primary cause of death by two physicians separately. A third expert physician determined the cause of death in case of any discrepancies. If a final diagnosis could not be made for any reason, the cause of death was recorded as “unknown”. In this study, we considered deaths due to CVD (ICD-10 codes I00–I99) and total cancer (ICD-10 codes C00–C97). Follow-up for this analysis continued until the subjects were lost to follow-up, death occurred, or the 15th of January 2021, whichever came first.

### 2.4. Statistical Analysis

Cox proportional hazards regression models were used for obtaining hazard ratios (HRs) and 95% confidence intervals (CIs). Aalen plots and the Schoenfeld residuals test confirmed the proportional hazards assumption for each mineral intake. The underlying temporal metric was age at the baseline. Multi-variable models were adjusted for baseline age (year), sex (male or female), smoking (yes or no), opium use (yes or no), BMI (kg/m^2^, continuous), wealth score (low, medium, high), physical activity level (low, intermediate, high), place of residence (urban or rural), ethnicity (Turkman or other), marital status (single, married), education (with or without formal education), history of hypertension (yes or no), and total energy intake (Kcal/day).

Multi-variate HRs were reported within quintiles, with the lowest quintiles used as the reference category. For the linear trend tests, the median value of each quintile was used. Also, the linear continuous variable of the intake of minerals was evaluated in Cox proportional hazards regression models. The HRs for the continuous scale were reported for each 100 mg/d increase in calcium, potassium, and magnesium intake, each 50 mg/d increase in phosphorus intake, each 1 mg/d increase in zinc, iron, and manganese intake, each 0.1 mg/d increase in copper intake, and each 10 µg/d increase in selenium intake (according to the IQR of the intakes of each mineral in the population). Additionally, we tested and plotted the association between each mineral and mortality using restricted cubic spline (RCS) functions. We utilized five knots and the median of the first quartile of intake as the reference point for each mineral in the RCS functions. The overall and nonlinear associations were evaluated using four and three *df* tests, respectively [15].

We also conducted interaction analyses by age, sex, BMI, and smoking, and none of them showed significant interactions. Due to the high prevalence of hypertension in this population (*n* = 6685, 15.97%), we did not exclude them from the main analyses; however, we conducted sensitivity analyses. The sensitivity analyses were performed by limiting the participants to those without a self-reported history of hypertension. We also re-performed the analyses excluding the subjects with reported alcohol consumption. Moreover, we made mutual adjustments for other elements (rather than the element of interest). The statistical analyses were carried out using the Stata software (version 14.1; StataCorp, College Station, TX, USA). Significant *p*-values were considered to be <0.05.

## 3. Results

In this study, a total of 41,863 subjects (42.6% male) with a total mean ± SD age at baseline of 51.46 ± 8.73 years were evaluated. During a median follow-up of 14.13 (IQR 1.64) years, 7217 deaths were recorded, with 39.2% (*n* = 2831) and 22.1% (*n* = 1595) of deaths related to CVD and cancer, respectively.

The baseline characteristics of the subjects in the GCS by quintiles of calcium intake per 1000 Kcal energy per day have been shown in Table 1. The participants in the highest quintile of calcium intake, compared to individuals in the lowest quintile, were significantly older, lived in urban settings, had formal education, were less likely to be smokers or opium users and more likely to be overweight, had higher wealth scores, and were physically active (<0.001). Among the minerals studied, only the mean calcium intakes in both genders and the mean zinc intakes in males were below the US Recommended Dietary Allowance (RDA) (Supplementary Materials, Table S1). We provided the correlation coefficients between the minerals in Supplementary Materials, Table S2.

### 3.1. All-Cause Mortality

**Figure 1:**
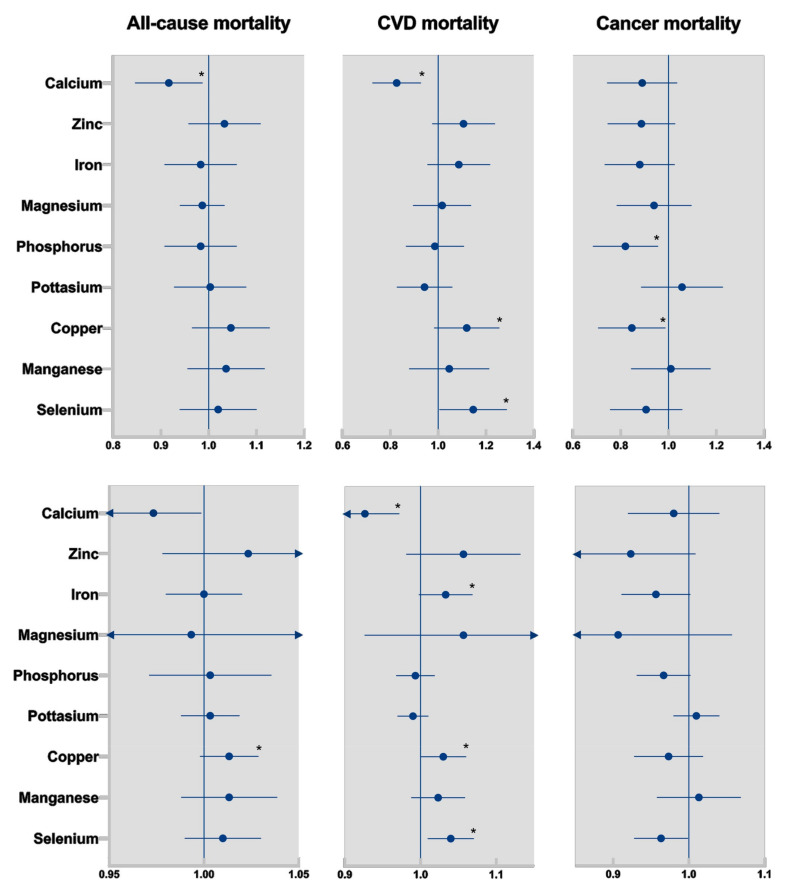
Hazard ratios and 95% confidence intervals of the highest quintile versus the lowest quintile of element intake (**Top**) and continuous (**Bottom**) in the multi-variable adjusted models. * represents a significant *p*-trend < 0.05.

Dietary calcium intake was inversely associated with all-cause mortality, comparing the highest quintile with the lowest quintile (HR_Q5 vs. Q1_ = 0.91, 95%CI = 0.85–0.99, *p*_trend_ < 0.05). For all the other minerals, there was no significant association between mineral intakes when comparing the highest quintile of intakes with the lowest quintile (Table 2). Using the continuous linear models, the mineral intakes were not associated with the risk of mortality (Figure 1, Table S3).

### 3.2. CVD Mortality

After adjustment for potential confounders, dietary calcium intake was inversely associated with CVD mortality when comparing the highest quintile of intake with the lowest quintile (HR_Q5 vs. Q1_ = 0.82, 95%CI = 0.73–0.93, *p*_trend_ < 0.01). In contrast, dietary selenium intake was positively associated with CVD mortality in the quintile model (HR_Q5 vs. Q1_ = 1.14, 95%CI = 1.01–1.29, *p*_trend_ < 0.05). More details are shown in Table 3. In the continuous linear models, as Figure 1 shows, calcium (per 100 mg/1000 Kcal/d increase) showed an inverse association (HR = 0.93, 95%CI = 0.88–0.97, *p*-value < 0.01), while iron (per 1 mg/1000 Kcal/d increase), copper (per 0.1 mg/1000 Kcal/d increase), and selenium (per 10 µg/1000 Kcal/d increase) were positively associated with CVD mortality (*p*-value < 0.05) (Table S4).

### 3.3. Cancer Mortality

Dietary phosphorus intake (HR_Q5 vs. Q1_ = 0.81, 95%CI = 0.69–0.96, *p*_trend_ < 0.05) and copper intake (HR_Q5 vs. Q1_ = 0.84, 95%CI = 0.71–0.99, *p*_trend_ < 0.05) were inversely associated with cancer mortality in the quintile models (Table 4). Moreover, there were no significant associations between mineral intakes and cancer mortality in the continuous models (Figure 1). More details on the continuous models have been provided in the Supplementary Materials (Table S5).

### 3.4. Restricted Cubic Splines

**Figure 2:**
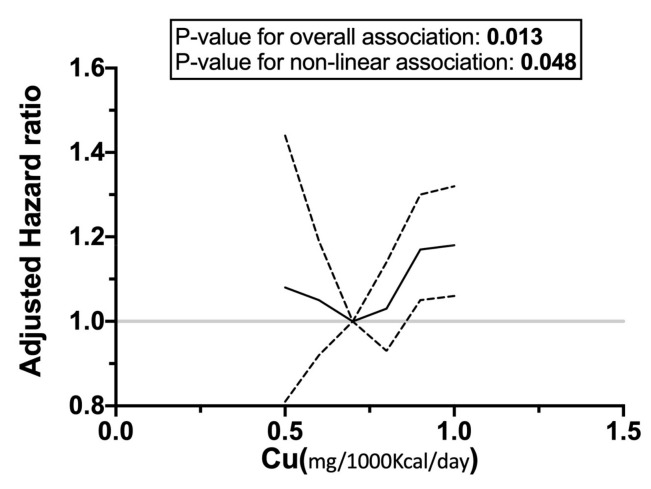

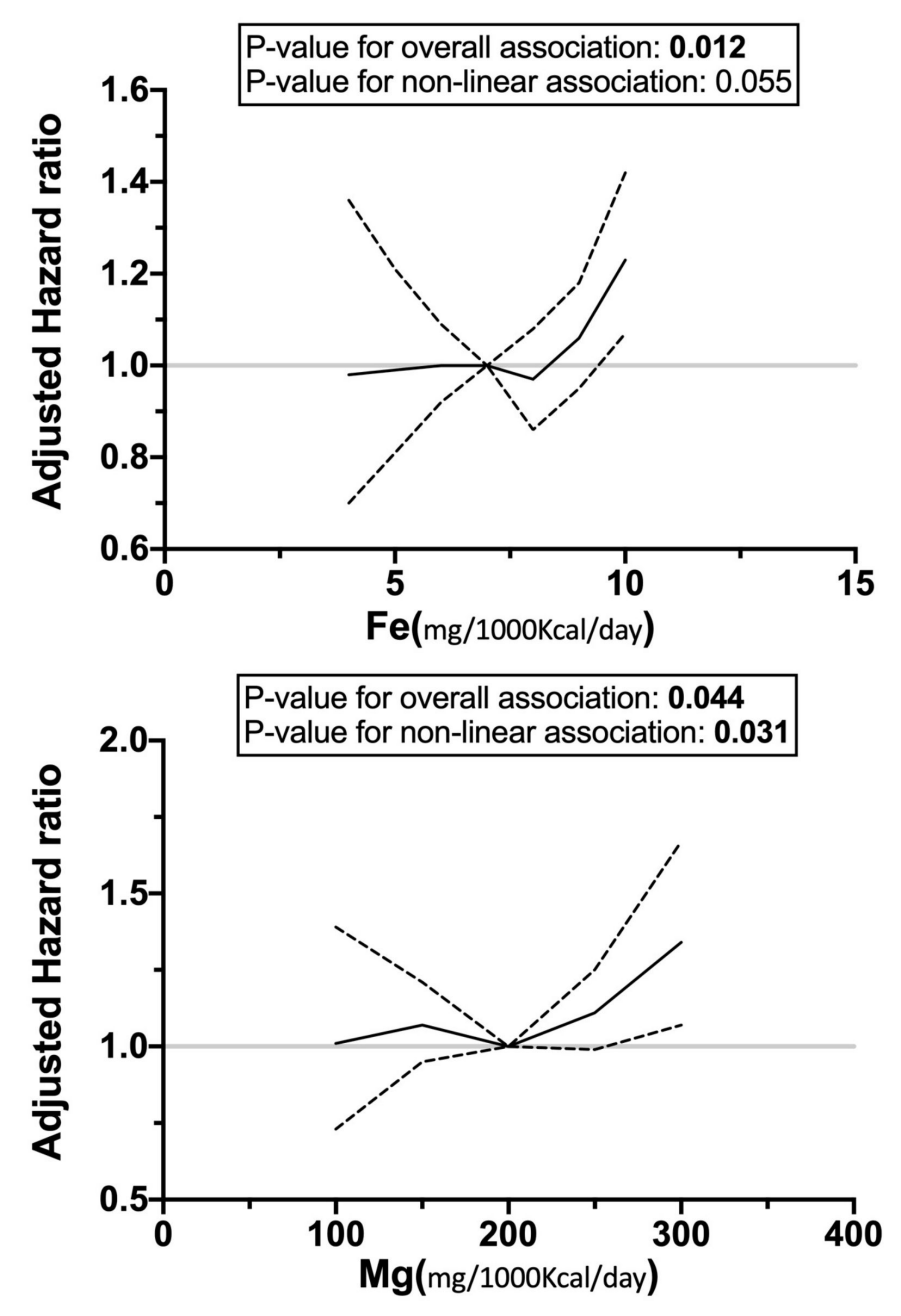
Associations between the intakes of copper, iron, and magnesium and cardiovascular mortality. Curved solid lines represent adjusted HRs, and dashed lines indicate their 95% CIs based on restricted cubic splines for specified amounts of intake for each mineral.

Among the minerals evaluated, the RCS models suggest U-shaped associations between the intakes of copper, magnesium, and iron and CVD mortality, but the *p* value for a nonlinear association was not significant for iron (Figure 2).

### 3.5. Sensitivity Analyses

In the non-hypertensive subjects (*n* = 35,178), there were no differences among significant associations between mineral intake and all-cause mortality in the quintile model compared to the results from the total subjects. Moreover, due to the very limited number of subjects with reported alcohol consumption (*n* = 1367, 3.27%), we did not adjust for drinking alcohol; however, we performed a sensitivity analysis. In the non-alcoholic subjects (*n* = 40,496), there were no differences among significant associations between mineral intake and all-cause mortality in the quintile models compared to the results from the total subjects. The mutual adjustment did not change our results significantly.

## 4. Discussion

We found that dietary calcium intake was inversely associated with all-cause mortality. Calcium intake was inversely associated, while selenium intake was positively associated with CVD mortality. Also, U-shaped associations were suggested for iron, copper, and magnesium and the risk of CVD mortality. Intakes of copper and phosphorus were also negatively associated with cancer mortality.

### 4.1. Calcium

Our findings show an inverse association between calcium intake and both all-cause and CVD mortality. This result has been previously reported in Sweden [16], Japan [17], China [18], and England [19]. Dietary calcium was linked to a notably decreased risk of all-cause mortality in a population of Swedish men [16]. Umesawa et al. observed a 27 and 23 percent decrease in the total CVD mortality of subjects with high vs. low calcium intake in Japanese men and women, respectively [17]. Also, Van der Pols et al. found that a high calcium intake in childhood is inversely linked to total mortality in adulthood and that a 65-year-long follow-up study of children in Britain found a decreased stroke mortality rate in children who had a family diet relatively high in calcium [19]. However, in some of the above studies, calcium intake was considered only from dairy products. Moreover, several studies found positive [20,21,22] or no such associations [23,24]. It seems that studies related to calcium intake and mortality are inconsistent. This discrepancy could be due to low- vs. high-fat dairy products as the main source of calcium in different populations. Asemi et al. [25], in a meta-analysis of observational studies, suggested that, for studies with a mean follow-up of 10 years or less, there is a substantial protective link between dietary calcium intake and all-cause and CVD mortality, while there is a significant relationship between calcium intake and an elevated risk of CVD mortality for studies with a longer follow-up. Furthermore, the mean calcium intakes for both genders in our study were below the RDA, potentially contributing to the stronger associations observed among the evaluated minerals.

There are some suggested mechanisms of action for the role of calcium in mortality. Through its impact on dyslipidemia and insulin resistance, a low calcium intake may increase the risk of CVD or mortality [26,27]. Calcium has a hypotensive impact, especially in people who consume higher amounts of sodium. Calcium also inhibits the aggregation of platelets [28,29]. Whey peptides, which are present in milk and dairy products, may have a hypotensive effect by inhibiting the angiotensin-converting enzyme in addition to possessing the health advantages of calcium, as milk and yogurt are the main sources of dietary calcium consumption for this population [30]. Furthermore, there is no correlation between calcium intake and vitamin D status in Iran since dairy products are not fortified with vitamin D, which is a significant issue in previous research evaluating the impact of calcium intake sources [31].

### 4.2. Iron

There was no association between iron intake and all-cause mortality, while previous studies mentioned a positive association between a higher iron intake and mortality. In a 12-year-long cohort study on US adults, it was indicated that subjects with a higher iron intake had a significantly increased all-cause mortality risk [32]. Moreover, Etemadi et al. suggested that the overall mortality risk increased with a higher dietary intake of heme iron [33]. In this population, the main source of iron was non-heme iron.

In this study, a suggestive U-shaped association between iron intake and CVD mortality was observed (Figure 2). Zhang et al. [34] suggested that dietary intake of total iron was positively associated with mortality from stroke and total CVD in Japanese men, and Lee et al. [35] indicated that a higher intake of heme iron might be related to CVD mortality in Iowa women. However, few studies suggested an inverse association between dietary iron intake and cardiovascular outcome [36]. Regarding cancer mortality and iron intake, in this study, we did not observe any significant association, which was in line with a previous study on Chinese adults [37].

Iron (heme and non-heme) intake can be involved in mortality via oxidative stress, which contributes to the aging process [38]. Heme iron has pro-oxidant properties that may encourage oxidative injury and inflammation in several organs [39]. Numerous health consequences, including diabetes, CVD, and cancer, have been linked to heme iron [40,41,42,43]. Lipid peroxidation and oxidative stress indicators can be brought on by dietary iron [44,45]. Low-density lipoprotein (LDL) can oxidize with the help of heme iron, perhaps acting as a catalyst. This can lead to inflammation that damages tissue and raises the risk of cardiovascular disease (CVD) [44].

### 4.3. Copper

In our study, a weak positive linear association between copper intake and CVD mortality was observed. The RCS models indicated a significant U-shaped association with CVD mortality. A J-shaped relationship between dietary copper intake and all-cause mortality in an adult Chinese population was demonstrated by Gan et al. in a recent study [46]. In a research on 58,646 healthy adults, Eshaka et al. investigated the relationship between dietary copper intake and the risk of death from CVD and found that it was positively linked with mortality from CVD in both genders [47], which was in line with our results. In contrast to our findings, Bates et al., in a study on British subjects, showed an inverse association with all-cause mortality [48]. They also reported an inverse association with cancer mortality that was not statistically significant, while a significant inverse association between copper intake and cancer mortality was observed in our study. In this study, the main sources of copper are bread and organ meats, while, in the Chinese study, grains and legumes were mentioned as the major sources. The other mentioned studies have not reported the main source of copper in their work.

Uncertain mechanisms underlie how copper relates to CVD mortality in distinct ways; however, copper oxidizes LDL cholesterol, increasing its atherogenicity [49]. By virtue of its association with the acute-phase-reactant ceruloplasmin, copper could also be regarded as an indicator of inflammatory risk [50,51].

### 4.4. Selenium

Dietary selenium intake in our study had a significant positive association with CVD mortality. No previous studies have suggested this positive association between selenium intake and CVD mortality. However, there are several studies evaluating selenium intake and mortality risks. Sun et al., in a study on 133,957 Chinese adults, suggested that dietary selenium intake was inversely associated with all-cause and CVD mortality in both genders but not cancer mortality [52]. They indicated that the highest quintile of selenium intake vs. the reference quintile was almost 20% less prone to all-cause and CVD mortality. In a recent study on US adults, it was shown that selenium intake was inversely associated with all-cause mortality [53]. Additionally, Xie et al. found a U-shaped association between selenium intake in a diet and death from all causes [54], which we did not observe in our results. Dietary adequacy could contribute to different results. The selenium intake in our study is much higher than the RDA (Supplementary Table S1), and this could cause a positive association between selenium intake and CVD mortality in this study. Also, a quite high level of selenium in the soil of this area may synergize the associations we found [55]. The main sources of selenium in this population are bread and egg.

Although there are several suggested roles of selenium in CVD, such as in limiting the oxidative alteration of lipids, decreasing platelet aggregation, lowering inflammation, and enhancing functional capillary recruitment [56,57,58], selenium, in combination with other antioxidants, has not been found to have a substantial protective impact on CVD or death in randomized trials [59,60]. Furthermore, it has been discovered in animal research that consuming too much selenium may increase DNA damage and cause oxidative damage [61,62,63].

### 4.5. Phosphorus

Phosphorus dietary intake had a significant inverse association with cancer mortality in our study. Chang et al. [64], evaluated dietary phosphorus intake and mortality in 9686 US adults and suggested that a higher phosphorus intake is associated with increased mortality in a healthy US population. The phosphorus density in their study was related to all-cause mortality at a phosphorus density amount >0.35 mg/kcal. Phosphorus density was also associated with cardiovascular mortality in the above-mentioned study. The source of phosphorous may contribute to the discrepancies observed. In our study, the main source of phosphorous was grains, while it could be dairy, processed food, or soft drinks in other populations.

### 4.6. Magnesium and Other Minerals

We found a suggestive U-shaped association between magnesium intake and cardiovascular mortality, while there are several studies with no association [16] or reduced [65,66,67,68] risk of mortality. We found no association between zinc or other mineral intake and all-cause and cause-specific mortality, which is in line with previous studies [69,70,71].

### 4.7. Strengths and Limitations

The strengths of our study included the use of a large sample size with a long duration of follow-up (median: 14.1 y), high retention rates, and detailed information on the participants’ diet. A significant number of elements’ intakes (10 elements) have been evaluated with all-cause, CVD, and cancer mortality, which makes the findings of this study unique. Also, another strength of our study was the restriction of our sample to a healthy population to minimize confounding by reverse causation. Additionally, by analyzing the data with energy adjustments, it is possible to gain the most insight possible into the relationships between dietary mineral intake and mortality. In this study, we used the density model for adjusting each element intake. We used three models (quintile, continuous, and RCS) to evaluate the association between elements and mortality, which was carefully adjusted for various plausible confounders.

Our study has some limitations that should be considered. First, the bioavailability of minerals is a concern. This is because inorganic elements may be more bioavailable than organic sources: for example, phosphate additives are more bioavailable (90–100% in inorganic vs. 40–60% in organic). Also, it has been suggested that dietary-derived elements from inorganic sources could have a more significant effect on serum concentrations [64,72,73]. Additionally, assessments of serum or plasma element levels are advised to obtain a better estimate of element intake, because the element levels in meals may differ between geographic regions due to the composition of the soil and water where the food was grown. Additionally, there might have been a measurement error because the nutrient databases we used may have underestimated the element concentration of food products heavy in inorganic element additions [74]. Also, dietary element intakes assessed at the baseline may not reflect recent dietary exposure as the intakes of said elements might have been changed during the long follow-up period. Moreover, it should be noted that our study conducted multiple comparisons, which may have affected the interpretation of our results.

Last, there remains the possibility of residual confounding. Some of the variables were self-reported: for example, the history of hypertension. Any high/low element intake may represent other aspects of unhealthy dietary patterns, which can lead to unhealthy outcomes [75]. Even after adjusting for various cofactors, unmeasured confounders such as access to healthcare might still have caused confounding.

## 5. Conclusions

In conclusion, calcium was inversely associated with all-cause and CVD mortality. Iron, copper, and magnesium showed a suggestive U-shaped association with CVD mortality. Selenium intakes were in positive associations with CVD mortality. Moreover, phosphorus and copper intakes were inversely associated with cancer mortality. In future research, longitudinal studies with repeated measures of element intakes and serum concentrations in different populations are needed to validate the findings. We suggest that future studies explicitly reference the primary sources of each mineral, considering that other components present in these foods may impact the outcomes and contribute to making studies comparable.

## Figures and Tables

**Table 1:** Baseline characteristics of the subjects (*n* = 41,863) in the Golestan Cohort Study by quintiles of calcium intake per 1000 Kcal energy per day and total population.

	Total Population	Calcium Quintiles					*p*-Value
		1	2	3	4	5	
Calcium, mg/1000 kcal/day	321.68 ± 85.03	222.08 ± 26.90	272.99 ± 10.63	309.26 ± 10.59	352.46 ± 15.24	451.12 ± 69.61	**<0.001**
Energy Intake (Kcal/day)	2160 ± 558	2065 ± 594	2159 ± 538	2206 ± 535	2220 ± 543	2151 ± 565	**<0.001**
Age, Years	51.46 ± 8.73	51.62 ± 8.76	51.01 ± 8.58	51.03 ± 8.48	51.33 ± 8.71	52.3 3 ± 9.05	**<0.001**
Sex, Male	17,870 (42.69)	3135 (37.56)	3544 (42.38)	3733 (44.51)	3788 (45.15)	3670 (43.81)	**<0.001**
Current smoker	7136 (17.05)	1457 (17.46)	1479 (17.69)	1467 (17.49)	1445 (17.22)	1288 (15.37)	**<0.001**
Opium user	6838 (16.33)	1834 (21.97)	1525 (18.24)	1326 (15.81)	1193 (14.22)	960 (11.46)	**<0.001**
BMI (Kg/m^2^)	26.47 ± 5.40	25.40 ± 5.38	26.15 ± 5.31	26.53 ± 5.38	26.93 ± 5.40	27.32 ± 5.33	**<0.001**
Wealth score							**<0.001**
Low	16,041 (38.32)	4800 (57.51)	3759 (44.95)	3025 (36.07)	2448 (29.18)	2009 (23.98)	
Medium	12,092 (28.88)	2222 (26.62)	2586 (30.93)	2548 (30.38)	2502 (29.82)	2234 (26.67)	
High	13,730 (32.80)	1325 (15.87)	2017 (24.17)	2813 (33.54)	3440 (41.0)	4135 (49.36)	
Physical Activity (MET)							**<0.001**
Low	13,044 (32.42)	2739 (34.53)	2539 (31.68)	2463 (30.44)	2555 (31.57)	2748 (33.90)	
Intermediate	13,250 (32.93)	2363 (29.79)	2550 (31.82)	2661 (32.88)	2790 (34.47)	2886 (35.60)	
High	13,945 (34.66)	2831 (35.69)	2926 (36.51)	2968 (36.68)	2748 (33.96)	2472 (30.50)	
Rural place of residence	33,730 (80.57)	7677 (91.97)	7328 (87.63)	6885 (82.10)	6363 (75.84)	5447 (65.37)	**<0.001**
Turkman Ethnicity	31,486 (75.21)	6154 (73.73)	6308 (75.44)	6352 (75.75)	6465 (77.06)	6207 (74.09)	**<0.001**
Married Status	37,045 (88.65)	7014 (84.21)	7389 (88.58)	7543 (90.08)	7574 (90.40)	7525 (89.94)	**<0.001**
No formal education	29,022 (69.33)	6815 (81.65)	6188 (74.00)	5710 (68.09)	5375 (64.06)	4934 (58.89)	**<0.001**
History of hypertension	6685 (15.97)	1295 (15.51)	1262 (15.09)	1280 (15.26)	1374 (16.38)	1474 (17.59)	**<0.001**

All the results are reported as mean ± standard deviation or number (%). Significant *p*-values were bolded. BMI = body mass index; MET = metabolic equivalent for task.

**Table 2:** HRs (95% CIs) of all-cause mortality by quintile of mineral intake.

		Quintile					*p*-Value
**Elements**		**1**	**2**	**3**	**4**	**5**	**Trend**
	**Calcium**						
	Intake (mg/1000 Kcal/day)	222.08 ± 26.90	272.99 ± 10.63	309.26 ± 10.59	352.46 ± 15.24	451.12 ± 69.61	**0.031**
	Person years	113,424	114,506	116,035	116,885	117,841	
	Cases, *n*	1648	1437	1350	1350	1432	
	Unadjusted HR	Ref	**0.86 (0.80–0.92) ^a^**	**0.79 (0.73–0.85) ^a^**	**0.78 (0.72–0.83) ^a^**	**0.80 (0.75–0.86) ^a^**	
	Age- and Sex-adjusted HR	Ref	**0.86 (0.83–0.96) ^b^**	**0.80 (0.75–0.86) ^a^**	**0.76 (0.70–0.81) ^a^**	**0.72 (0.67–0.77) ^a^**	
	Multi-variable-adjusted HR	Ref	0.94 (0.88–1.02)	0.93 (0.86–1.00)	**0.91 (0.84–0.98) ^c^**	**0.91 (0.85–0.99) ^c^**	
	**Zinc**						
	Intake (mg/1000 Kcal/day)	3.86 ± 0.29	4.32 ± 0.08	4.57 ± 0.06	4.83 ± 0.08	5.33 ± 0.36	0.253
	Person years	116,557	116,502	116,105	115,380	114,148	
	Cases, *n*	1556	1356	1350	1392	1563	
	Unadjusted HR	Ref	**0.88 (0.81–0.94) ^b^**	**0.88 (0.82–0.94) ^b^**	**0.91 (0.85–0.98) ^c^**	1.04 (0.97–1.12)	
	Age- and Sex-adjusted HR	Ref	**0.92 (0.85–0.99) ^c^**	**0.91 (0.85–0.98) ^c^**	0.93 (0.87–1.00)	0.96 (0.90–1.03)	
	Multi-variable-adjusted HR	Ref	0.97 (0.90–1.05)	0.97 (0.90–1.05)	1.00 (0.93–1.08)	1.03 (0.96–1.11)	
	**Iron**						
	Intake (mg/1000 Kcal/day)	6.23 ± 0.63	7.30 ± 0.18	7.86 ± 0.14	8.40 ± 0.17	9.33 ± 0.53	0.722
	Person years	116,246	116,471	116,155	115,389	114,431	
	Cases, *n*	1597	1417	1359	1357	1487	
	Unadjusted HR	Ref	**0.89 (0.83–0.95) ^b^**	**0.86 (0.80–0.92) ^a^**	**0.86 (0.80–0.93) ^a^**	0.96 (0.89–1.03)	
	Age- and Sex-adjusted HR	Ref	0.96 (0.89–1.03)	0.94 (0.87–1.01)	0.99 (0.92–1.06)	1.01 (0.94–1.08)	
	Multi-variable-adjusted HR	Ref	0.98 (0.90–1.05)	0.96 (0.89–1.04)	0.97 (0.90–1.05)	0.98 (0.91–1.06)	
	**Magnesium**						
	Intake (mg/1000 Kcal/day)	163.20 ± 17.50	192.96 ± 5.03	208.32 ± 4.11	223.47 ± 4.86	249.63 ± 15.84	0.475
	Person years	115,220	116,729	116,688	115,944	114,111	
	Cases, *n*	1558	1357	1323	1381	1598	
	Unadjusted HR	Ref	**0.85 (0.79–0.92) ^a^**	**0.83 (0.77–0.90) ^a^**	**0.88 (0.82–0.95) ^b^**	1.04 (0.97–1.12)	
	Age- and Sex-adjusted HR	Ref	**0.91 (0.85–0.98) ^c^**	**0.89 (0.82–0.95) ^b^**	0.95 (0.88–1.02)	1.05 (0.97–1.12)	
	Multi-variable-adjusted HR	Ref	**0.92 (0.85–0.99) ^c^**	**0.90 (0.84–0.98) ^c^**	0.93 (0.86–1.00)	0.96 (0.89–1.04)	
	**Phosphorus**						
	Intake (mg/1000 Kcal/day)	508.09 ± 41.18	572.14 ± 11.23	607.38 ± 9.52	642.78 ± 11.50	710.50 ± 46.25	0.647
	Person years	115,949	115,658	115,715	115,409	115,961	
	Cases, *n*	1517	1414	1390	1376	1520	
	Unadjusted HR	Ref	0.94 (0.87–1.01)	**0.92 (0.86–0.99) ^c^**	**0.91 (0.85–0.98) ^c^**	1.00 (0.93–1.07)	
	Age- and Sex-adjusted HR	Ref	0.94 (0.88–1.01)	**0.91 (0.84–0.97) ^c^**	**0.88 (0.81–0.94) ^b^**	**0.86 (0.80–0.93) ^a^**	
	Multi-variable-adjusted HR	Ref	0.99 (0.92–1.07)	0.99 (0.91–1.06)	0.97 (0.89–1.05)	0.98 (0.91–1.06)	
	**Potassium**						
	Intake (mg/1000 Kcal/day)	1086.39 ± 71.36	1204.04 ± 22.82	1281.61 ± 22.92	1373.97 ± 32.33	1597.58 ± 179.11	0.405
	Person years	115,763	116,108	115,930	115,719	115,172	
	Cases, *n*	1491	1348	1352	1435	1591	
	Unadjusted HR	Ref	**0.90 (0.84–0.97) ^b^**	**0.91 (0.84–0.97) ^c^**	0.96 (0.89–1.03)	1.07 (0.99–1.14)	
	Age- and Sex-adjusted HR	Ref	**0.92 (0.85–0.99) ^c^**	**0.92 (0.85–0.99) ^c^**	**0.92 (0.86–0.99) ^c^**	0.99 (0.92–1.06)	
	Multi-variable-adjusted HR	Ref	0.93 (0.86–1.00)	0.93 (0.87–1.01)	0.96 (0.89–1.04)	1.00 (0.93–1.08)	
	**Copper**						
	Intake (mg/1000 Kcal/day)	0.64 ± 0.04	0.71 ± 0.01	0.76 ± 0.1	0.80 ± 0.01	0.93 ± 0.16	0.297
	Person years	116,094	116,396	116,237	115,665	114,300	
	Cases, *n*	1497	1369	1367	1433	1551	
	Unadjusted HR	Ref	**0.91 (0.85–0.98) ^c^**	**0.92 (0.85–0.99) ^c^**	0.97 (0.90–1.04)	1.06 (0.99–1.14)	
	Age- and Sex-adjusted HR	Ref	0.98 (0.91–1.05)	0.99 (0.92–1.06)	1.01 (0.94–1.09)	**1.10 (1.02–1.18) ^b^**	
	Multi-variable-adjusted HR	Ref	0.98 (0.90–1.05)	0.98 (0.90–1.05)	0.97 (0.90–1.04)	1.06 (0.96–1.12)	
	**Manganese**						
	Intake (mg/1000 Kcal/day)	2.96 ± 0.47	3.79 ± 0.14	4.26 ± 0.12	4.75 ± 0.16	5.75 ± 0.77	0.337
	Person years	116,690	116,977	116,353	115,909	112,763	
	Cases, *n*	1431	1306	1382	1399	1699	
	Unadjusted HR	Ref	**0.91 (0.84–0.98) ^c^**	0.97 (0.90–1.05)	0.99 (0.92–1.07)	**1.25 (1.17–1.35) ^c^**	
	Age- and Sex-adjusted HR	Ref	0.96 (0.89–1.04)	1.02 (0.94–1.09)	1.04 (0.96–1.12)	**1.23 (1.15–1.32) ^a^**	
	Multi-variable-adjusted HR	Ref	0.94 (0.87–1.02)	0.98 (0.91–1.06)	0.93 (0.86–1.01)	1.03 (0.96–1.12)	
	**Selenium**						
	Intake (mg/1000 Kcal/day)	49.54 ± 7.19	62.28 ± 2.21	68.99 ± 1.75	75.30 ± 1.98	86.20 ± 6.30	0.366
	Person years	116,703	116,773	116,093	115,596	113,567	
	Cases, *n*	1463	1397	1396	1391	1570	
	Unadjusted HR	Ref	0.96 (0.89–1.03)	0.97 (0.90–1.04)	0.97 (0.90–1.05)	**1.13 (1.05–1.21) ^a^**	
	Age- and Sex-adjusted HR	Ref	0.99 (0.92–1.07)	1.02 (0.95–1.10)	1.02 (0.95–1.10)	**1.11 (1.03–1.19) ^b^**	
	Multi-variable-adjusted HR	Ref	0.99 (0.91–1.07)	1.03 (0.96–1.12)	1.02 (0.95–1.11)	1.02 (0.94–1.10)	

Data presented as the hazard ratio (95%CI). *p*-value ^a^ < 0.001, ^b^ < 0.01, ^c^ < 0.05. Significant HRs were bolded. The multi-variable models were adjusted for age (year), sex (male or female), place of residence (urban or rural), education (with or without formal education), married status (yes or no), ethnicity (Turkman or others), history of hypertension, BMI, physical activity level (low, intermediate, high), opium use (yes or no), smoking (yes or no), energy (Kcal), and wealth score (low, medium, high). The *p* trend has been reported for the multi-variable adjusted model. Elements are bolded for enhanced clarity and ease of understanding.

**Table 3:** HRs (95% CIs) of cardiovascular mortality by quintile of mineral intake.

		Quintile					*p*-Value
		**1**	**2**	**3**	**4**	**5**	**Trend**
**Elements**	**Calcium**						
	Intake (mg/1000 Kcal/day)	222.08 ± 26.90	272.99 ± 10.63	309.26 ± 10.59	352.46 ± 15.24	451.12 ± 69.61	**0.003**
	Person years	113,424	114,506	116,035	116,885	117,841	
	Cases, *n*	672	554	549	515	541	
	Unadjusted HR	Ref	**0.81 (0.72–0.91) ^a^**	**0.79 (0.70–0.88) ^a^**	**0.73 (0.65–0.82) ^a^**	**0.75 (0.67–0.84) ^a^**	
	Age- and Sex-adjusted HR	Ref	**0.85 (0.76–0.95) ^b^**	**0.81 (0.72–0.91) ^a^**	**0.71 (0.64–0.80) ^a^**	**0.67 (0.59–0.75) ^a^**	
	Multi-variable-adjusted HR	Ref	**0.88 (0.78–0.99) ^c^**	0.92 (0.82–1.04)	**0.84 (0.74–0.95) ^b^**	**0.82 (0.73–0.93) ^b^**	
	**Zinc**						
	Intake (mg/1000 Kcal/day)	3.86 ± 0.29	4.32 ± 0.08	4.57 ± 0.06	4.83 ± 0.08	5.33 ± 0.36	0.073
	Person years	116,557	116,502	116,105	115,380	114,148	
	Cases, *n*	583	518	519	553	658	
	Unadjusted HR	Ref	**0.81 (0.72–0.92) ^b^**	**0.82 (0.73–0.92) ^b^**	**0.84 (0.75–0.95) ^b^**	**1.12 (1.00–1.25) ^c^**	
	Age- and Sex-adjusted HR	Ref	0.94 (0.84–1.06)	0.94 (0.84–1.06)	1.00 (0.89–1.12)	1.08 (0.96–1.20)	
	Multi-variable-adjusted HR	Ref	1.00 (0.88–1.13)	0.98 (0.86–1.11)	1.04 (0.92–1.18)	1.10 (0.98–1.24)	
	**Iron**						
	Intake (mg/1000 Kcal/day)	6.23 ± 0.63	7.30 ± 0.18	7.86 ± 0.14	8.40 ± 0.17	9.33 ± 0.53	0.191
	Person years	116,246	116,471	116,155	115,389	114,431	
	Cases, *n*	609	532	510	531	649	
	Unadjusted HR	Ref	**0.87 (0.78–0.98) ^c^**	**0.84 (0.75–0.95) ^c^**	0.89 (0.79–1.00)	1.10 (0.98–1.23)	
	Age- and Sex-adjusted HR	Ref	0.95 (0.85–1.07)	0.94 (0.83–1.05)	1.03 (0.91–1.16)	**1.17 (1.05–1.31) ^b^**	
	Multi-variable-adjusted HR	Ref	0.95 (0.84–1.07)	0.94 (0.83–1.06)	0.98 (0.86–1.10)	1.08 (0.96–1.22)	
	**Magnesium**						
	Intake (mg/1000 Kcal/day)	163.20 ± 17.50	192.96 ± 5.03	208.32 ± 4.11	223.47 ± 4.86	249.63 ± 15.84	0.837
	Person years	115,220	116,729	116,688	115,944	114,111	
	Cases, *n*	613	510	513	520	675	
	Unadjusted HR	Ref	**0.81 (0.72–0.92) ^b^**	**0.82 (0.73–0.92) ^b^**	**0.84 (0.75–0.95) ^b^**	**1.12 (1.00–1.25) ^c^**	
	Age- and Sex-adjusted HR	Ref	0.88 (0.79–1.00)	0.89 (0.79–1.00)	0.92 (0.82–1.04)	**1.14 (1.02–1.27) ^b^**	
	Multi-variable-adjusted HR	Ref	**0.87 (0.77–0.99) ^c^**	0.92 (0.81–1.04)	0.88 (0.78–1.00)	1.01 (0.90–1.14)	
	**Phosphorus**						
	Intake (mg/1000 Kcal/day)	508.09 ± 41.18	572.14 ± 11.23	607.38 ± 9.52	642.78 ± 11.50	710.50 ± 46.25	0.626
	Person years	115,949	115,658	115,715	115,409	115,961	
	Cases, *n*	581	560	540	537	613	
	Unadjusted HR	Ref	0.97 (0.86–1.09)	0.93 (0.83–1.05)	0.93 (0.83–1.05)	1.05 (0.94–1.18)	
	Age- and Sex-adjusted HR	Ref	0.98 (0.87–1.10)	0.92 (0.82–1.03)	0.89 (0.79–1.00)	0.90 (0.81–1.01)	
	Multi-variable-adjusted HR	Ref	0.99 (0.88–1.12)	0.97 (0.86–1.10)	0.95 (0.84–1.07)	0.98 (0.87–1.11)	
	**Potassium**						
	Intake (mg/1000 Kcal/day)	1086.39 ± 71.36	1204.04 ± 22.82	1281.61 ± 22.92	1373.97 ± 32.33	1597.58 ± 179.11	0.772
	Person years	115,763	116,108	115,930	115,719	115,172	
	Cases, *n*	597	520	511	585	618	
	Unadjusted HR	Ref	**0.87 (0.77–0.98) ^c^**	**0.85 (0.76–0.96) ^c^**	0.98 (0.87–1.10)	1.03 (0.92–1.16)	
	Age- and Sex-adjusted HR	Ref	0.89 (0.79–1.00)	**0.87 (0.77–0.98) ^c^**	0.93 (0.83–1.05)	0.94 (0.84–1.06)	
	Multi-variable-adjusted HR	Ref	0.89 (0.79–1.01)	0.88 (0.78–1.00)	0.96 (0.85–1.09)	0.94 (0.83–1.06)	
	**Copper**						
	Intake (mg/1000 Kcal/day)	0.64 ± 0.04	0.71 ± 0.01	0.76 ± 0.1	0.80 ± 0.01	0.93 ± 0.16	**0.016**
	Person years	116,094	116,396	116,237	115,665	114,300	
	Cases, *n*	582	500	497	586	666	
	Unadjusted HR	Ref	**0.86 (0.76–0.97) ^a^**	**0.86 (0.76–0.96) ^a^**	1.02 (0.91–1.14)	**1.17 (1.05–1.31) ^b^**	
	Age- and Sex-adjusted HR	Ref	0.92 (0.82–1.04)	0.93 (0.83–1.05)	1.08 (0.96–1.21)	**1.23 (1.10–1.38) ^a^**	
	Multi-variable-adjusted HR	Ref	0.91 (0.81–1.03)	0.91 (0.81–1.04)	0.99 (0.87–1.11)	1.11 (0.99–1.26)	
	**Manganese**						
	Intake (mg/1000 Kcal/day)	2.96 ± 0.47	3.79 ± 0.14	4.26 ± 0.12	4.75 ± 0.16	5.75 ± 0.77	0.293
	Person years	116,690	116,977	116,353	115,909	112,763	
	Cases, *n*	566	502	544	526	693	
	Unadjusted HR	Ref	0.88 (0.78–1.01)	0.97 (0.86–1.09)	0.94 (0.84–1.06)	**1.29 (1.16–1.44) ^a^**	
	Age- and Sex-adjusted HR	Ref	0.94 (0.84–1.07)	1.03 (0.91–1.16)	1.00 (0.88–1.12)	**1.29 (1.15–1.44) ^a^**	
	Multi-variable-adjusted HR	Ref	0.92 (0.81–1.04)	0.99 (0.87–1.12)	0.90 (0.79–1.02)	1.07 (0.87–1.20)	
	**Selenium**						
	Intake (mg/1000 Kcal/day)	49.54 ± 7.19	62.28 ± 2.21	68.99 ± 1.75	75.30 ± 1.98	86.20 ± 6.30	**0.037**
	Person years	116,703	116,773	116,093	115,596	113,567	
	Cases, *n*	554	539	534	523	681	
	Unadjusted HR	Ref	0.98 (0.87–1.10)	0.98 (0.87–1.10)	0.97 (0.86–1.09)	**1.29 (1.15–1.44) ^a^**	
	Age- and Sex-adjusted HR	Ref	1.02 (0.90–1.15)	1.05 (0.93–1.18)	1.03 (0.91–1.16)	**1.28 (1.14–1.43) ^a^**	
	Multi-variable-adjusted HR	Ref	1.01 (0.89–1.14)	1.05 (0.93–1.19)	1.00 (0.88–1.14)	**1.14 (1.01–1.29) ^c^**	

Data presented as the hazard ratio (95%CI). *p*-value ^a^ < 0.001 ^b^ < 0.01 ^c^ < 0.05. Significant HRs were bolded. The multi-variable models were adjusted for age (year), sex (male or female), place of residence (urban or rural), education (with or without formal education), married status (yes or no), ethnicity (Turkman or others), history of hypertension, BMI, physical activity level (low, intermediate, high), opium use (yes or no), smoking (yes or no), energy (Kcal), and wealth score (low, medium, high). The *p* trend has been reported for the multi-variable adjusted model. Elements are bolded for enhanced clarity and ease of understanding.

**Table 4:** HRs (95% CIs) of cancer mortality by quintile of mineral intake.

		Quintile					*p*-Value
**Elements**		**1**	**2**	**3**	**4**	**5**	**Trend**
	**Calcium**						
	Intake (mg/1000 Kcal/day)	222.08 ± 26.90	272.99 ± 10.63	309.26 ± 10.59	352.46 ± 15.24	451.12 ± 69.61	0.132
	Person years	113,424	114,506	116,035	116,885	117,841	
	Cases, *n*	355	336	285	311	308	
	Unadjusted HR	Ref	0.93 (0.80–1.08)	**0.78 (0.66–0.91) ^b^**	**0.84 (0.72–0.98) ^c^**	**0.82 (0.70–0.95) ^c^**	
	Age- and Sex-adjusted HR	Ref	0.95 (0.82–1.10)	**0.77 (0.66–0.90) ^b^**	**0.81 (0.69–0.94) ^b^**	**0.74 (0.63–0.86) ^a^**	
	Multi-variable-adjusted HR	Ref	0.972 (0.83–1.14)	**0.84 (0.71–0.99) ^c^**	0.91 (0.77–1.07)	0.88 (0.75–1.04)	
	**Zinc**						
	Intake (mg/1000 Kcal/day)	3.86 ± 0.29	4.32 ± 0.08	4.57 ± 0.06	4.83 ± 0.08	5.33 ± 0.36	0.085
	Person years	116,557	116,502	116,105	115,380	114,148	
	Cases, *n*	371	332	293	293	303	
	Unadjusted HR	Ref	0.89 (0.77–1.04)	**0.79 (0.68–0.92) ^b^**	**0.81 (0.69–0.94) ^b^**	**0.84 (0.72–0.97) ^c^**	
	Age- and Sex-adjusted HR	Ref	0.92 (0.79–1.07)	**0.81 (0.69–0.94) ^b^**	**0.81 (0.69–0.94) ^b^**	**0.78 (0.67–0.91) ^b^**	
	Multi-variable-adjusted HR	Ref	0.95 (0.81–1.11)	0.86 (0.73–1.01)	0.88 (0.75–1.03)	0.88 (0.75–1.03)	
	**Iron**						
	Intake (mg/1000 Kcal/day)	6.23 ± 0.63	7.30 ± 0.18	7.86 ± 0.14	8.40 ± 0.17	9.33 ± 0.53	0.109
	Person years	116,246	116,471	116,155	115,389	114,431	
	Cases, *n*	355	331	323	296	290	
	Unadjusted HR	Ref	0.93 (0.80–1.08)	0.91 (0.78–1.06)	**0.84 (0.72–0.98) ^c^**	**0.83 (0.71–0.97) ^c^**	
	Age- and Sex-adjusted HR	Ref	0.97 (0.84–1.13)	0.96 (0.83–1.12)	0.92 (0.78–1.07)	0.85 (0.73–1.00)	
	Multi-variable-adjusted HR	Ref	0.97 (0.83–1.14)	0.95 (0.81–1.11)	0.92 (0.78–1.08)	0.87 (0.74–1.03)	
	**Magnesium**						
	Intake (mg/1000 Kcal/day)	163.20 ± 17.50	192.96 ± 5.03	208.32 ± 4.11	223.47 ± 4.86	249.63 ± 15.84	0.263
	Person years	115,220	116,729	116,688	115,944	114,111	
	Cases, *n*	332	334	300	304	325	
	Unadjusted HR	Ref	0.99 (0.85–1.15)	0.89 (0.76–1.04)	0.90 (0.77–1.06)	0.99 (0.85–1.15)	
	Age- and Sex-adjusted HR	Ref	1.02 (0.88–1.19)	0.91 (0.78–1.06)	0.93 (0.80–1.09)	0.96 (0.82–1.12)	
	Multi-variable-adjusted HR	Ref	1.01 (0.86–1.18)	0.86 (0.73–1.01)	0.93 (0.79–1.09)	0.93 (0.79–1.10)	
	**Phosphorus**						
	Intake (mg/1000 Kcal/day)	508.09 ± 41.18	572.14 ± 11.23	607.38 ± 9.52	642.78 ± 11.50	710.50 ± 46.25	**0.022**
	Person years	115,949	115,658	115,715	115,409	115,961	
	Cases, *n*	348	342	294	330	281	
	Unadjusted HR	Ref	0.98 (0.85–1.14)	**0.84 (0.72–0.99) ^c^**	0.95 (0.82–1.11)	**0.80 (0.69–0.94) ^b^**	
	Age- and Sex-adjusted HR	Ref	0.98 (0.85–1.14)	**0.83 (0.71–0.97) ^c^**	0.91 (0.78–1.06)	**0.71 (0.60–0.83) ^a^**	
	Multi-variable-adjusted HR	Ref	1.01 (0.87–1.18)	0.89 (0.76–1.05)	1.00 (0.85–1.17)	**0.81 (0.69–0.96) ^c^**	
	**Potassium**						
	Intake (mg/1000 Kcal/day)	1086.39 ± 71.36	1204.04 ± 22.82	1281.61 ± 22.92	1373.97 ± 32.33	1597.58 ± 179.11	0.648
	Person years	115,763	116,108	115,930	115,719	115,172	
	Cases, *n*	331	330	296	298	340	
	Unadjusted HR	Ref	0.99 (0.85–1.15)	0.89 (0.76–1.04)	0.90 (0.77–1.05)	1.03 (0.88–1.20)	
	Age- and Sex-adjusted HR	Ref	1.00 (0.86–1.17)	0.90 (0.77–1.06)	0.88 (0.75–1.03)	0.99 (0.85–1.15)	
	Multi-variable-adjusted HR	Ref	0.99 (0.85–1.16)	0.92 (0.78–1.08)	0.93 (0.79–1.09)	1.05 (0.89–1.23)	
	**Copper**						
	Intake (mg/1000 Kcal/day)	0.64 ± 0.04	0.71 ± 0.01	0.76 ± 0.011	0.80 ± 0.01	0.93 ± 0.16	**0.028**
	Person years	116,094	116,396	116,237	115,665	114,300	
	Cases, *n*	355	325	321	297	297	
	Unadjusted HR	Ref	0.91 (0.78–1.06)	0.90 (0.77–1.05)	**0.84 (0.72–0.98) ^c^**	**0.85 (0.73–0.99) ^c^**	
	Age- and Sex-adjusted HR	Ref	0.95 (0.82–1.11)	0.94 (0.81–1.09)	**0.85 (0.73–0.99) ^c^**	**0.84 (0.72–0.98) ^c^**	
	Multi-variable-adjusted HR	Ref	0.92 (0.79–1.08)	0.93 (0.79–1.08)	0.85 (0.72–1.00)	**0.84 (0.71–0.99) ^c^**	
	**Manganese**						
	Intake (mg/1000 Kcal/day)	2.96 ± 0.47	3.79 ± 0.14	4.26 ± 0.12	4.75 ± 0.16	5.75 ± 0.77	0.628
	Person years	116,690	116,977	116,353	115,909	112,763	
	Cases, *n*	322	276	318	317	362	
	Unadjusted HR	Ref	0.85 (0.72–1.00)	0.99 (0.85–1.15)	0.99 (0.85–1.16)	**1.17 (1.01–1.36) ^c^**	
	Age- and Sex-adjusted HR	Ref	0.87 (0.74–1.03)	1.00 (0.85–1.17)	1.00 (0.85–1.17)	1.12 (0.96–1.31)	
	Multi-variable-adjusted HR	Ref	**0.83 (0.70–0.98) ^c^**	0.95 (0.81–1.12)	0.89 (0.76–1.05)	1.00 (0.85–1.18)	
	**Selenium**						
	Intake (mg/1000 Kcal/day)	49.54 ± 7.19	62.28 ± 2.21	68.99 ± 1.75	75.30 ± 1.98	86.20 ± 6.30	0.307
	Person years	116,703	116,773	116,093	115,596	113,567	
	Cases, *n*	319	330	328	313	305	
	Unadjusted HR	Ref	1.03 (0.88–1.20)	1.03 (0.89–1.21)	0.99 (0.85–1.16)	0.99 (0.84–1.16)	
	Age- and Sex-adjusted HR	Ref	1.04 (0.89–1.22)	1.05 (0.90–1.23)	1.01 (0.86–1.18)	0.95 (0.81–1.11)	
	Multi-variable-adjusted HR	Ref	0.98 (0.84–1.15)	1.02 (0.87–1.20)	0.99 (0.84–1.16)	0.90 (0.76–1.06)	

Data presented as the hazard ratio (95%CI). *p*-value ^a^ < 0.001 ^b^ < 0.01 ^c^ < 0.05. Significant HRs were bolded. The multi-variable models were adjusted for age (year), sex (male or female), place of residence (urban or rural), education (with or without formal education), married status (yes or no), ethnicity (Turkman or others), BMI, physical activity level (low, intermediate, high), opium use (yes or no), smoking (yes or no), energy (Kcal), and wealth score (low, medium, high). The *p* trend has been reported for the multi-variable adjusted model. Elements are bolded for enhanced clarity and ease of understanding.

## Data Availability

The datasets used and/or analyzed during the current study are available from the corresponding author upon request.

## Supplementary Materials

The following supporting information can be downloaded at https://www.mdpi.com/article/10.3390/nu16030344/s1: Figure S1. Participants’ flow chart; Table S1. mean dietary intake, recommended dietary allowances, and adequate intakes of elements; Table S2. Correlation coefficients between minerals (*n* = 41,863); Table S3. HRs (95% CIs) of all-cause mortality for continuous mineral intake; Table S4. HRs (95% CIs) of cardiovascular mortality for continuous mineral intake; and Table S5. HRs (95% Cis) of cancer mortality for continuous mineral intake.
