# Supplementary material for: Mineral Intake and Cardiovascular Disease, Cancer, and All-Cause Mortality: Findings from the Golestan Cohort Study
Source: Nutrients. 2024 Jan 24;16(3):344. doi: 10.3390/nu16030344 (PMC10857363; doi:10.3390/nu16030344)
Supplement: Supplementary file 1 [file nutrients-16-00344-s001.zip › Supplementary file-Jan7.pdf]

Table S1. Mean dietary intake of minerals in the Golestan Cohort Study and corresponding Recommended Dietary Allowances (RDA).

**Elements**	**Male**		**Female**	
	Mean dietary intake (SD)	RDA*	Mean dietary intake (SD)	RDA*
Zinc (mg/d)	10.8±2.8	11	9.2±2.5	8
Iron (mg/d)	18.5±5.0	8	15.7±4.5	8
Calcium (mg/d)	765.3±269.2	1,000	644.8±239.2	1,200
Magnesium (mg/d)	493.4±133.4	420	414.4±120.5	320
Copper (mg/d)	1.86±0.5	0.9	1.52±0.4	0.9
Selenium (µg/d)	164.2±48.9	55	136.6±44.2	55

*RDA: Recommended Dietary Allowances of minerals (for age group 51-70, as the mean of our population was 53 years old) Edited from: National Academies of Sciences, Engineering, and Medicine; Health and Medicine Division; Food and Nutrition Board; Committee to Review the Dietary Reference Intakes for Sodium and Potassium; Oria M, Harrison M, Stallings VA, editors. Dietary Reference Intakes for Sodium and Potassium. Washington (DC): National Academies Press (US); 2019 Mar 5. Appendix J, Dietary Reference Intakes Summary Tables. Available from: https://www.ncbi.nlm.nih.gov/books/NBK545442/

Table S2. Correlation coefficients between minerals(n=41,863).

	Calcium	Zinc	Iron	Magnesium	Phosphorus	Potassium	Copper	Manganese	Selenium
**Calcium**	1								
**Zinc**	0.315	1							
**Iron**	-0.080	0.468	1						
**Magnesium**	0.030	0.427	0.760	1					
**Phosphorus**	0.648	0.792	0.371	0.470	1				
**Potassium**	0.254	0.157	-0.096	0.177	0.199	1			
**Copper**	0.061	0.358	0.500	0.477	0.249	0.335	1		
**Manganese**	-0.206	0.119	0.512	0.801	0.079	0.274	0.357	1	
**Selenium**	-0.102	0.592	0.855	0.757	0.496	-0.278	0.340	0.501	1

All reported numbers are correlation coefficients. All of the coefficients were statistically significant ($P<0.001$).

Table S3. HRs (95% CIs) of all-cause mortality for continuous mineral intake.

	Unadjusted HR	P-value	Age and sex adjusted HR	P-value	Multi-variable adjusted HR	P-value
Calcium, per 100-mg/1000Kcal/d increase	**0.93(0.91-0.96)**	**<0.001**	**0.88(0.86-0.91)**	**<0.001**	0.97(0.95-1.00)	0.140
Zinc, per 1-mg/1000Kcal/d increase	**1.04(1.00-1.09)**	**0.032**	0.99(0.94-1.03)	0.642	1.02(0.98-1.07)	0.201
Iron, per 1-mg/1000Kcal/d increase	0.99(0.97-1.01)	0.430	1.01(0.99-1.03)	0.265	1.00(0.98-1.02)	0.716
Magnesium, per 100-mg/1000Kcal/d increase	**1.11(1.03-1.19)**	**0.006**	**1.09(1.02-1.18)**	**0.011**	0.99(0.92-1.07)	0.933
Phosphorus, per 50-mg/1000Kcal/d increase	1.00(0.98-1.01)	0.861	**0.96(0.95-0.98)**	**<0.001**	0.99(0.98-1.01)	0.947
Potassium, per 100-mg/1000Kcal/d increase	**1.01(1.00-1.03)**	**0.002**	1.00(0.99-1.01)	0.442	1.00(0.99-1.02)	0.163
Copper, per 0.1-mg/1000Kcal/d increase	1.01(0.99-1.03)	0.056	**1.02(1.01-1.04)**	**0.001**	**1.01(1.00-1.03)**	**0.047**
Manganese, per 1-mg/1000Kcal/d increase	**1.10(1.07-1.12)**	**<0.001**	**1.08(1.06-1.10)**	**<0.001**	1.01(0.99-1.04)	0.148
Selenium, per 10-µg/1000Kcal/d increase	**1.03(1.01-1.05)**	**<0.001**	**1.02(1.01-1.04)**	**0.001**	1.01(0.99-1.03)	0.228

Data presented as the Hazard Ratio (95%CI). Significant HRs were bolded.
Multivariable models were adjusted for age (year), sex (male or female), place of residence (urban or rural), education (with or without formal education), married status (yes or no), ethnicity (Turkman or others), history of hypertension, BMI, physical activity level (low, intermediate, high), opium use (yes or no), smoking (yes or no), energy (Kcal), wealth score (low, medium, high).

Table S4. HRs (95% CIs) of cardiovascular mortality for continuous mineral intake.

	Unadjusted HR	P-value	Age and sex adjusted HR	P-value	Multi-variable adjusted HR	P-value
Calcium, per 100-mg/1000Kcal/d increase	**0.89(0.85-0.94)**	**<0.001**	**0.85(0.81-0.89)**	**<0.001**	**0.93(0.88-0.97)**	**0.004**
Zinc, per 1-mg/1000Kcal/d increase	**1.12(1.05-1.20)**	**<0.001**	1.05(0.98-1.12)	0.101	1.06(0.98-1.13)	0.099
Iron, per 1-mg/1000Kcal/d increase	**1.04(1.00-1.07)**	**0.018**	**1.06(1.02-1.09)**	**<0.001**	**1.03(1.00-1.07)**	**0.031**
Magnesium, per 100-mg/1000Kcal/d increase	**1.22(1.08-1.37)**	**<0.001**	**1.21(1.08-1.36)**	**<0.001**	1.05(0.93-1.19)	0.372
Phosphorus, per 50-mg/1000Kcal/d increase	1.01(0.98-1.03)	0.378	0.97(0.95-1.00)	0.053	0.99(0.97-1.02)	0.766
Potassium, per 100-mg/1000Kcal/d increase	1.01(0.99-1.03)	0.159	0.99(0.97-1.01)	0.710	0.99(0.97-1.01)	0.760
Copper, per 0.1-mg/1000Kcal/d increase	**1.03(1.00-1.06)**	**0.014**	**1.04(1.02-1.07)**	**<0.001**	**1.03(1.00-1.06)**	**0.027**
Manganese, per 1-mg/1000Kcal/d increase	**1.11(1.07-1.14)**	**<0.001**	**1.09(1.06-1.13)**	**<0.001**	1.02(0.99-1.06)	0.141
Selenium, per 10-µg/1000Kcal/d increase	**1.07(1.04-1.10)**	**<0.001**	**1.06(1.04-1.09)**	**<0.001**	**1.04(1.01-1.07)**	**0.008**

Data presented as the Hazard Ratio (95%CI). Significant HRs were bolded.

Multivariable models were adjusted for age (year), sex (male or female), place of residence (urban or rural), education (with or without formal education), married status (yes or no), ethnicity (Turkman or others), history of hypertension, BMI, physical activity level (low, intermediate, high), opium use (yes or no), smoking (yes or no), energy (Kcal), wealth score (low, medium, high).

Table S5. HRs (95% CIs) of cancer mortality for continuous mineral intake.

	Unadjusted HR	P-value	Age and sex adjusted HR	P-value	Multi-variable adjusted HR	P-value
Calcium, per 100-mg/1000Kcal/d increase	0.95(0.89-1.00)	0.094	**0.90(0.85-0.95)**	**0.001**	0.98(0.92-1.04)	0.527
Zinc, per 1-mg/1000Kcal/d increase	**0.91(0.82-0.99)**	**0.045**	**0.87(0.79-0.95)**	**0.003**	0.92(0.84-1.01)	0.100
Iron, per 1-mg/1000Kcal/d increase	**0.94(0.90-0.98)**	**0.011**	**0.95(0.91-0.99)**	**0.039**	0.96(0.91-1.00)	0.097
Magnesium, per 100-mg/1000Kcal/d increase	0.96(0.82-1.13)	0.687	0.93(0.80-1.09)	0.404	0.90(0.76-1.06)	0.222
Phosphorus, per 50-mg/1000Kcal/d increase	0.96(0.93-1.00)	0.052	**0.94(0.91-0.97)**	**<0.001**	0.97(0.93-1.00)	0.089
Potassium, per 100-mg/1000Kcal/d increase	1.00(0.98-1.03)	0.600	0.99(0.97-1.02)	0.970	1.01(0.98-1.04)	0.329
Copper, per 0.1-mg/1000Kcal/d increase	0.97(0.93-1.01)	0.253	0.97(0.93-1.01)	0.210	0.97(0.93-1.02)	0.294
Manganese, per 1-mg/1000Kcal/d increase	**1.07(1.02-1.13)**	**<0.001**	**1.05(1.01-1.10)**	**<0.015**	1.01(0.96-1.07)	0.460
Selenium, per 10-µg/1000Kcal/d increase	0.99(0.95-1.02)	0.611	0.97(0.94-1.01)	0.245	0.96(0.93-1.00)	0.114

Data presented as the Hazard Ratio (95%CI). Significant HRs were bolded.

Multivariable models were adjusted for age (year), sex (male or female), place of residence (urban or rural), education (with or without formal education), married status (yes or no), ethnicity (Turkman or others), BMI, physical activity level (low, intermediate, high), opium use (yes or no), smoking (yes or no), energy (Kcal), wealth score (low, medium, high).
