# Supplementary figures and images for: Mineral Intake and Cardiovascular Disease, Cancer, and All-Cause Mortality: Findings from the Golestan Cohort Study
Source: Nutrients. 2024 Jan 24;16(3):344. doi: 10.3390/nu16030344 (PMC10857363; doi:10.3390/nu16030344)

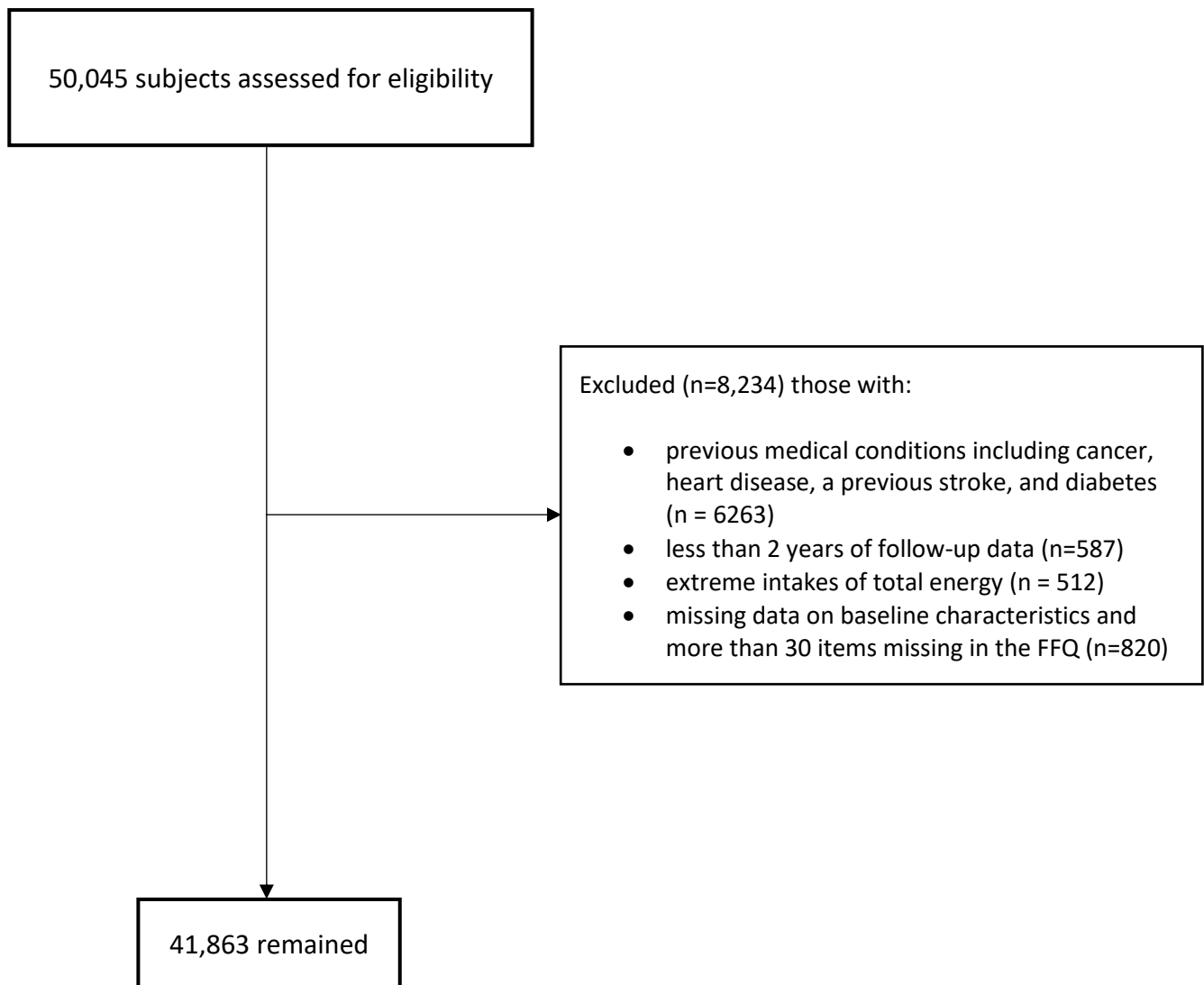

Figure S1. Participants flow chart

Supplement: Supplementary file 1 [file nutrients-16-00344-s001.zip › Figure S1-Jan7.pdf]
